# Supplementary material for: Knockdown of TIM3 Hampers Dendritic Cell Maturation and Induces Immune Suppression by Modulating T-Cell Responses
Source: Int J Mol Sci. 2025 May 2;26(9):4332. doi: 10.3390/ijms26094332 (PMC12072576; doi:10.3390/ijms26094332)
Supplement: Supplementary file 1 [file ijms-26-04332-s001.zip › ijms-3537577-supplementary.pdf]

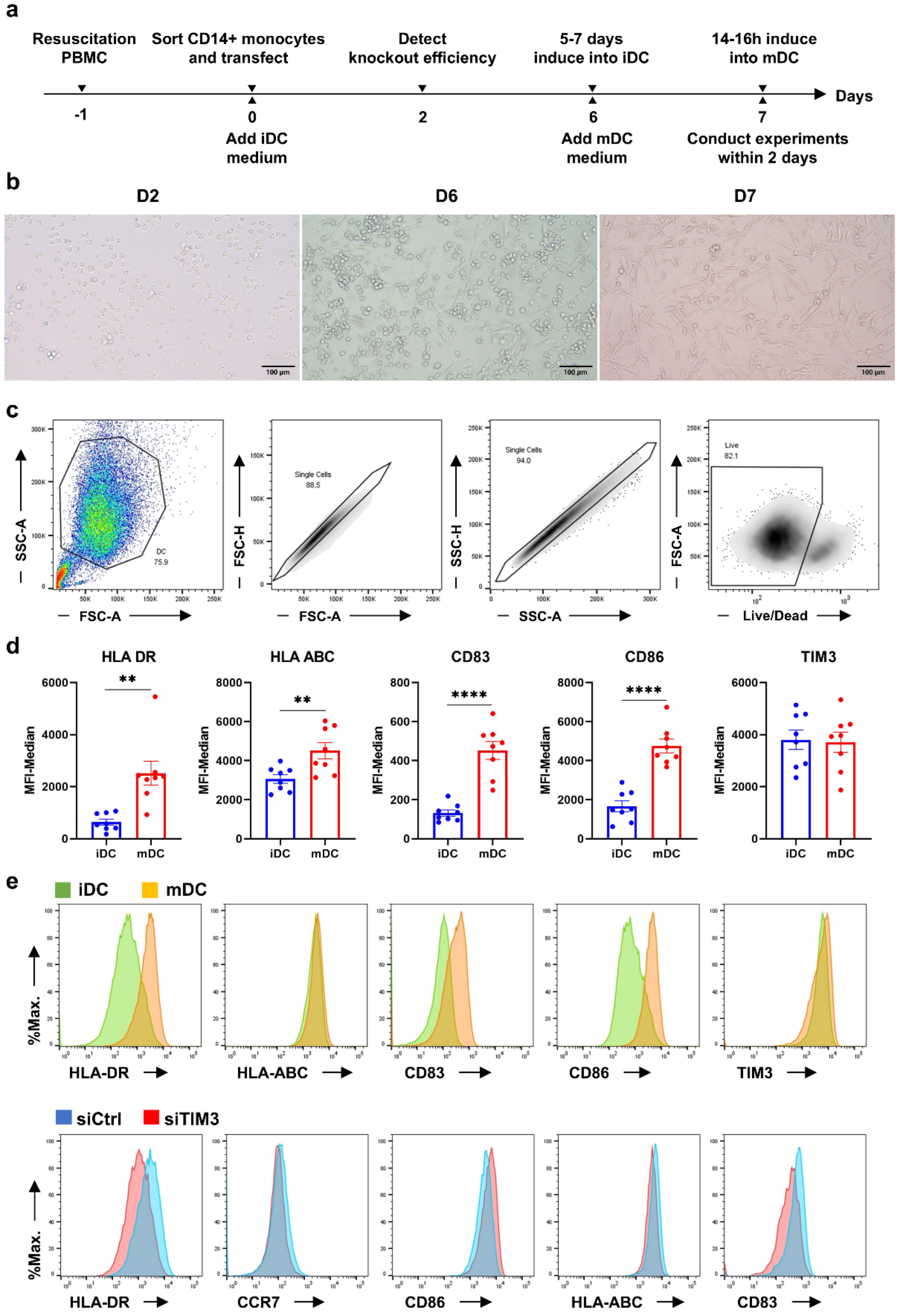

Figure S1. Phenotypic alterations during in vitro maturation of DCs. (a) Diagram of the protocol for generating mDCs from human PBMCs. iDC, immature dendritic cell. mDC, mature dendritic cell. (b) Representative bright-field images demonstrating the morphological characteristics of adherent-cultured DCs. (c) Gating strategy for FC analysis of DCs. (d) FC analysis of surface marker expression (HLA-DR, HLA-ABC, CD83, CD86, and TIM3) during differentiation from iDCs to mDCs. Data (n = 8) are shown as

mean ± SEM. Paired t-test (**p < 0.01; ****p < 0.0001; ns: not significant). (e) MFI distribution profiles of surface markers in DCs (upper panel: mDCs and iDCs; lower panel: siCtrl and siTIM3).

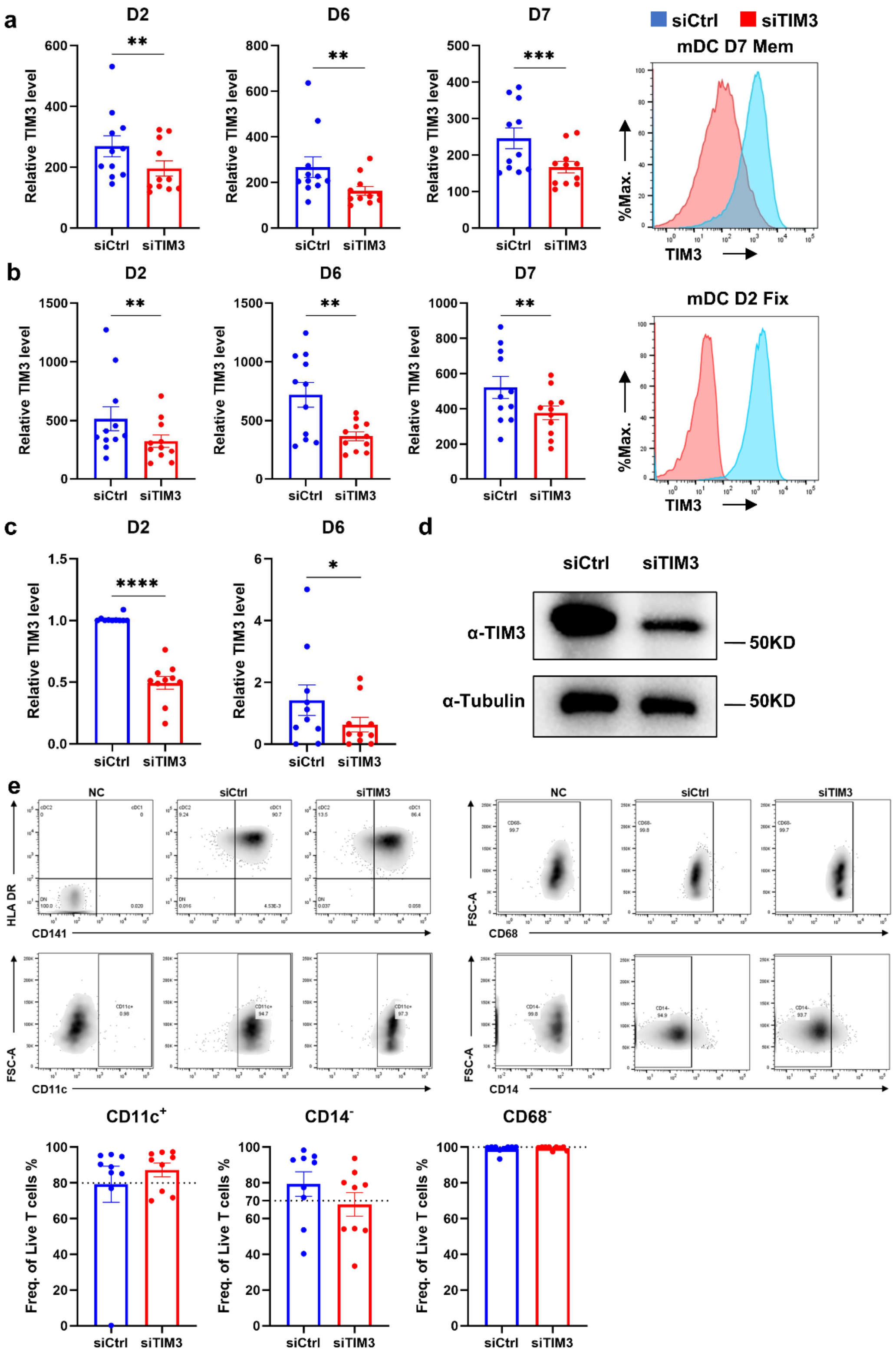

Figure S2. Efficiency of siRNA interference with TIM3 expression in DCs. Quantification of (a) surface and (b) fixed (n=11) TIM3 expression of DCs on day 2, 6, and 7 after intervention. Right panel: Representative FC histograms showing the MFI distribution profiles of TIM3-silenced DCs. (c) qPCR

(n=10, three replicates) was used to analyze the suppression effect at days 2 and 6. (d) Representative WB images of TIM3 protein levels in DCs at 72 h post siRNA transfection (n=1, three replicates). α-TIM3, anti-TIM3; α-tubulin, anti-tubulin. (e) The subtypes and proportions of cDC1 in total mDCs by FC (n=9). Data are presented as mean ± SEM. *p<0.05; ****p<0.0001. Paired t-tests.

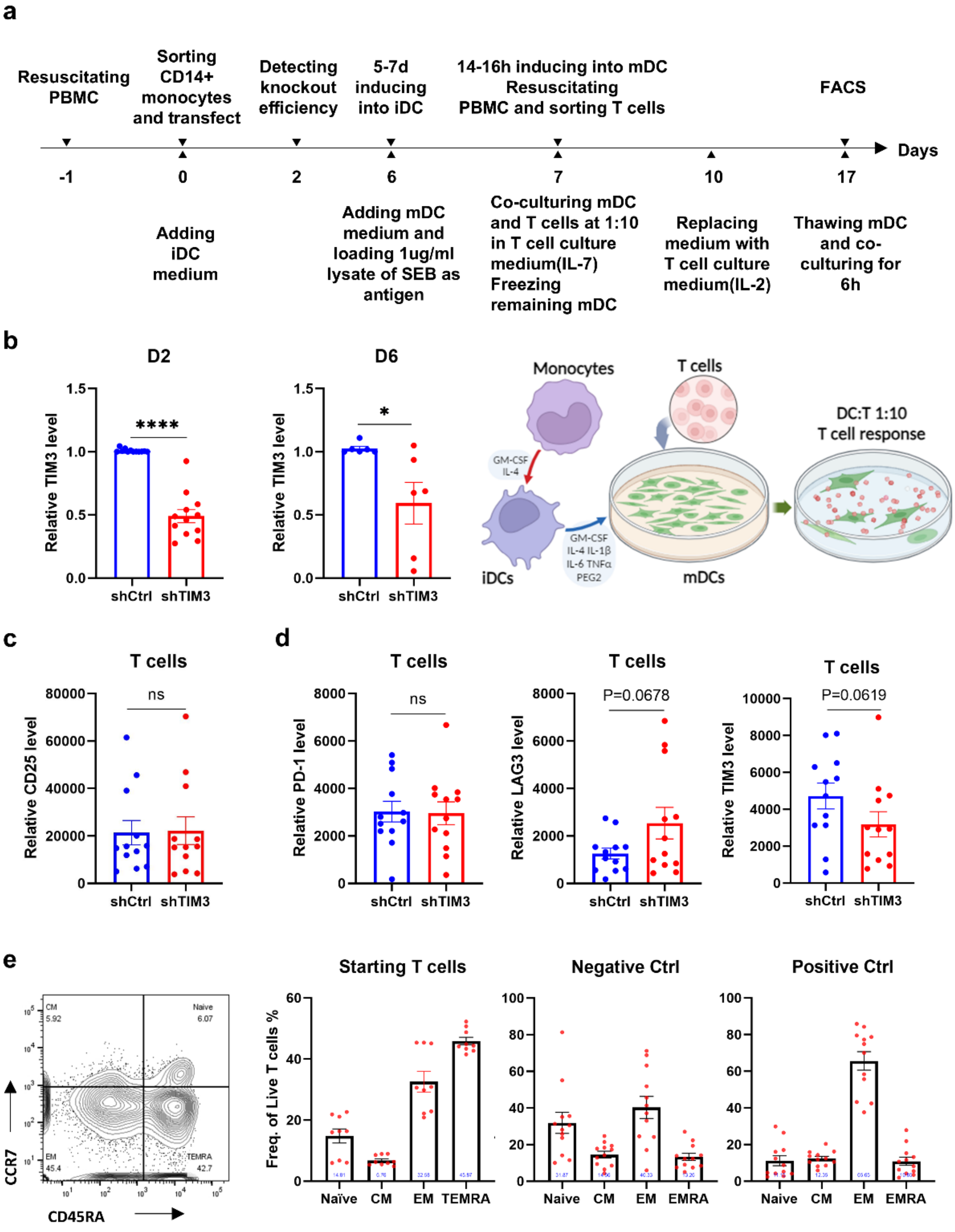

Figure S3. Functional analysis of DC–T cell interactions. (a) Schematic representation of the DC–T cell response process. (b) qPCR analysis of TIM3 knockdown efficiency mediated by shRNA at days 2 (n = 12, three replicates) and 6 (n = 6, three replicates). (c-d) FC evaluation of (c) CD25 expression and (d) immune checkpoint marker profiles on T cells (n = 12). (e) Left to right: Gating strategy for T-cell maturation status based on CD45RA and CCR7 expression; Pre-co-culture T-cell maturation baseline (n = 9); Negative control (T cells cultured alone for 10 days, n = 12); Positive control (T cells stimulated with αCD3/CD28 for 10 days, n = 12). All donors were treated and compared equally with NC shRNA and

TIM3 shRNA. Data are presented as mean ± SEM. Paired t-tests. ns, not significant; *p < 0.05; ****p < 0.0001. *Culture conditions:* T-cell culture medium (IL-7): RPMI-1640 with 10% FBS and 10 ng/mL rhIL-7; T-cell culture medium (IL-2): RPMI-1640 with 10% FBS, 10 ng/mL rhIL-7, and 50 U/mL rhIL-2.

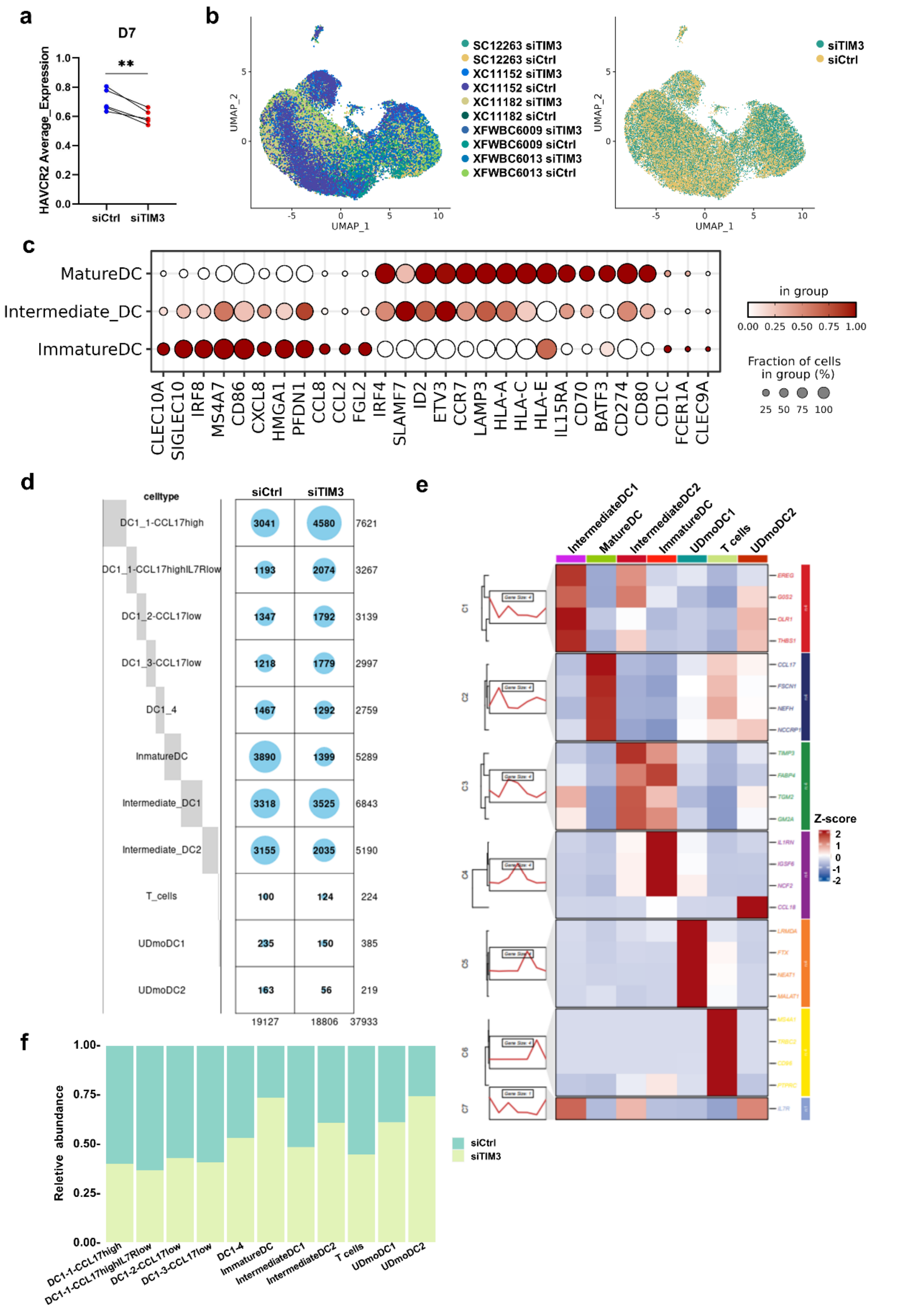

Figure S4. Single-cell transcriptomic profiling of siCtrl and siTIM3 DCs. (a) Average *HAVCR2* (TIM3) expression in mDCs at day 7, quantified using scRNA-seq. (b) Umap projection of 37,933 single cells

from siRNA-treated samples, color-coded by sample type. (c) Dot plot displaying gene expression patterns distinguishing immature DCs, intermediate DCs, and mature DCs, also with markers for cDC1 and cDC2. (d) Proportional abundance bubble plot of major cell types. (e) Annotation of cell subclusters, DEGs, and expression trends in the dataset. (e) Bar chart of relative abundance in different subsets. N=5 healthy donors per group, and all donors were treated and compared using NC siRNA and TIM3 siRNA equally. Data are presented as mean ± SEM. Paired t-test. **p<0.01.

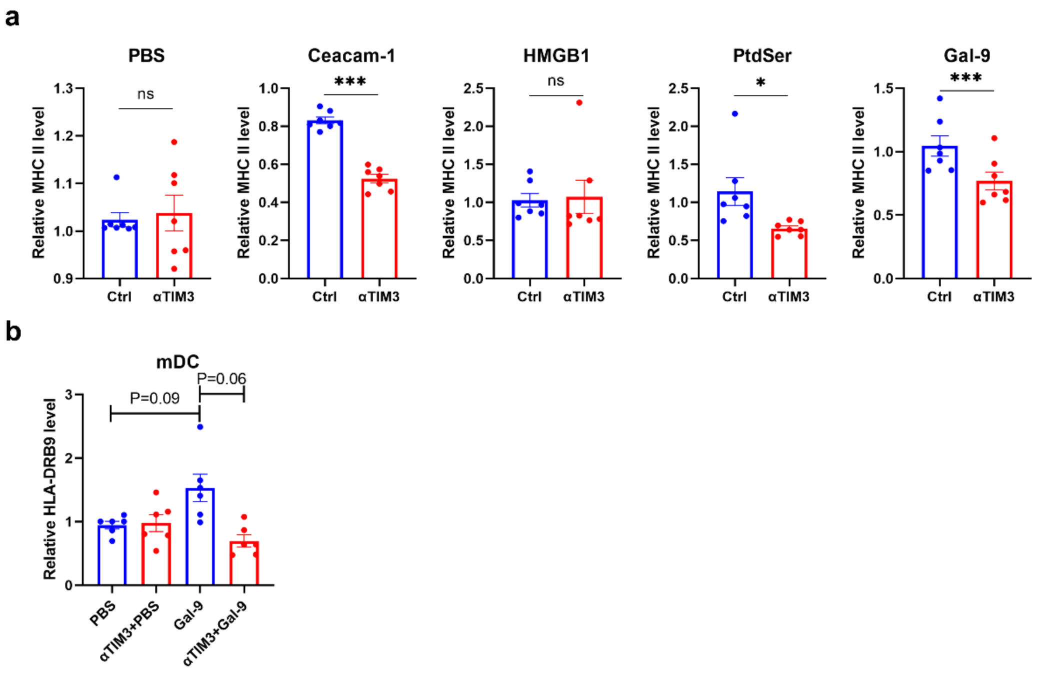

Figure S5. TIM3 antibody blockade suppresses ligand-induced MHC class II expression in mDCs. (a) mDCs pre-treated overnight with 10 µg/mL α-TIM3 (sabatolimab) were stimulated with TIM3 ligands (CEACAM1, HMGB1, PtdSer, or Gal-9) separately. qPCR quantification of average expression levels of seven MHC class II genes (*HLA-DRA, HLA-DRB9, HLA-DPA1, HLA-DPB1, HLA-DQA1, HLA-DQA2, HLA-DQB1*) is shown (n = 3 donors, three technical replicates). Data were analyzed using a paired Student's t-test; mean ± SEM. ns: not significant; **p < 0.05; ***p < 0.001. (b) qPCR analysis of HLA-DRB9 expression under four conditions: PBS control, α-TIM3-only blockade, Gal-9 stimulation alone, and Gal-9 stimulation post-α-TIM3 pretreatment (n = 6 donors, three technical replicates). Statistical comparison using one-way ANOVA; mean ± SEM. *Stimulation conditions*: Gal-9: 2 µg/mL for 30 min; HMGB1: 250 ng/mL for 2 h; CEACAM1: 10 µg/mL for 2 h; PtdSer: 20 µg/mL for 2 h; PBS: 2 h. *Blockade conditions*: mDCs were incubated with 10 µg/mL sabatolimab overnight in complete medium (37°C, 5% CO_2). Before stimulation, the medium was replaced with fresh sabatolimab-containing medium, followed by ligand addition.

Table S1. Key resource

Reagent or resource	Source	Identifier
Antibody		
BV786 anti-human CD279(PD-1) Antibody	BD Horizon	Cat# 563789
AF647 anti-human CD279(PD-1) Antibody	BD Horizon	Cat# 560838
BV786 anti-human CD152(CTLA-4) Antibody	BD Horizon	Cat# 563931
AF488 anti-human RORrt Antibody	BD Horizon	Cat# 563621
AF647 anti-GATA3 Antibody	BD Horizon	Cat# 560068
PE/CY7 anti-GATA3 Antibody	BD Horizon	Cat# 560405
PerCP/CY5.5 anti-Tbet Antibody	BD Horizon	Cat# 561316
PE anti-human CD11c Antibody	BD Pharmigen	Cat# 555392
APC/CY7 anti-human CD14 Antibody	BD Pharmigen	Cat# 557831
AF647 anti-human TIM3 Antibody	BD Pharmigen	Cat# 565558
PE/Dazzle™594 anti-human CD4 Antibody	Biolegend	Cat# 300548
PE/Dazzle™594 anti-human CD4 Antibody	Biolegend	Cat# 317448
PerCP/CY5.5 anti-human CD8 Antibody	Biolegend	Cat# 344710
FITC anti-human CD80 Antibody	Biolegend	Cat# 305206
PerCP/CY5.5 anti-human CD45RA Antibody	Biolegend	Cat# 304122
PE/CY7 anti-human HLA-DR Antibody	Biolegend	Cat# 307616
PE anti-human CD86 Antibody	Biolegend	Cat# 305406
APC anti-human HLA-A, B, C Antibody	Biolegend	Cat# 311410
PE anti-Tbet Antibody	Biolegend	Cat# 644810
APC/CY7 anti-human CD25 Antibody	BD Pharmigen	Cat# 557753
BB515 anti-human CD4 Antibody	BD Pharmigen	Cat# 564419
FITC anti-human CD8 Antibody	BD Pharmigen	Cat# 551347
PE/CF594 anti-human FoxP3 Antibody	BD Horizon	Cat# 562421
PE anti-human CCR7(CD197) Antibody	BD Pharmigen	Cat# 552176
AF647 anti-human LAG-3(CD223) Antibody	BD Pharmigen	Cat# 565716
BB700 anti-human CD86 Antibody	BD Pharmigen	Cat# 566473
APC-H7 anti-human CD80 Antibody	BD Pharmigen	Cat# 561134
FITC anti-human CD83 Antibody	BD Pharmigen	Cat# 556910
PE anti-human HLA DR Antibody	BD Pharmigen	Cat# 556644

APC anti-human CD1a Antibody	BD Pharmigen	Cat# 559775
PE anti-human CD1c Antibody	BD Pharmigen	Cat# 555392
APC/CY7 anti-human CD14 Antibody	BD Pharmigen	Cat# 557831
FITC anti-human CD68 Antibody	BD Pharmigen	Cat# 562117
AF746 anti-human TIM3 Antibody	BD Pharmigen	Cat# 565558
PE anti-human CD154 Antibody	Biolegend	Cat# 310805
APC anti-human CD69 Antibody	Biolegend	Cat# 310909
APC anti-human CD25 Antibody	Biolegend	Cat# 302609
FITC anti-human CD44 Antibody	BD Pharmigen	Cat# 555478
BV650 anti-human CD74 Antibody	BD Biosciences	Cat# 743734
LIVE/DEAD™ Fixable Blue Dead Cell Stain Kit	Invitrogen™	Cat# L23105
Purified NA/LE Mouse Anti-Human CD28	BD Pharmigen	Cat# 555725
CellTrace™ CFSE Cell Proliferation Kit	Invitrogen™	Cat# C34554
FcR Blocking Reagent, human	Miltenyi Biotec	Cat# 130-059-901
Fixable Viability Stain 510	BD Horizon™	Cat# 564406
Fixable Viability Stain 620	BD Horizon™	Cat# 564996
Anti-TIM 3 antibody [EPR22241] ab241332	Abcam	Cat# EPR22241
UltraPure™ DNase/RNase-Free Distilled Water	Gibco	Cat# 10977015
Chemicals, peptides, and proteins		
Recombinant Human IL-6 Protein	R&D Systems	Cat# 206-IL-200/CF
Prostaglandin E2	R&D Systems	Cat# 2296/10
Recombinant Human TNF-alpha Protein	R&D Systems	Cat# 210-TA
Recombinant Human GM-CSF Protein	R&D Systems	Cat# 215-GM
Recombinant Human IL-4 Protein, CF	R&D Systems	Cat# BT-004
Recombinant Human IL-1 beta/IL-1F2 Protein	R&D Systems	Cat# 201-LB
HiPerFect Transfection Reagent	Qiagen	Cat# 301705
CTS AIM V Medium	Gibco	Cat# 0870112-DK
Serum-Free Cell Freezing Medium (2X)	DAKEWE	Cat# 6032011
Lymphoprep™ Density gradient medium	Stemcell	Cat# 07851
Red cell lysis buffer	TIANGEN	Cat# RT122
Staphylococcal Enterotoxin B, highly purified	TOXIN Tech.	Cat# BT 202
3-sn-Phosphatidyl-L-serine	Merck	Cat# P7769-5MG
Human HMGB1 Protein, His Tag	ACROBiosystems	Cat# HM1-H5220-100ug

Human CEACAM-1/CD66a Protein, His Tag	ACROBiosystems	Cat# CE1-H5220-100ug
Cholesterol	MCE	Cat# HY-N0322
Human Galectin-9 / LGALS9 Protein, His Tag	ACROBiosystems	Cat# LG9-H5244-50ug
Critical commercial assays		
EasySep™ Human T Cell Isolation Kit	Stemcell	Cat# 17951
EasySep™ Human CD14 Positive Selection Kit II	Stemcell	Cat# 17858
Cytometric Bead Array (CBA) Human Th1/Th2/Th17 CBA Kit	BD™	Cat# 560484
eBioscience™ Foxp3 / Transcription Factor Staining Buffer Set	Invitrogen	Cat# 00-5523-00
Human CCL17/TARC ELISA Kit	Multi Sciences	Cat# EK1115-96
Human CCL18/PARC ELISA Kit	Multi Sciences	Cat# EK1148-96
Total RNA Kit Ⅱ (50)	Omega	Cat# R6934-01
TB Green® Premix Ex Taq™ II (Tli RNase H Plus)	Takara	Cat# RR820A
PrimeScript™ RT Reagent Kit (Perfect Real Time)	Takara	Cat# RR037A
BD Rhapsody™ Enhanced Cartridge Reagent Kit	BD™	Cat# 664887
BD Rhapsody™ Cartridge Kit	BD™	Cat# 633733
BD Rhapsody™ cDNA Kit	BD™	Cat# 633773
BD Rhapsody™ Whole Transcriptome Analysis (WTA) Amplification Kit	BD™	Cat# 633801
BD™ Hu Single Cell Sample Multiplexing Kit	BD™	Cat# 633781

Table S2. RT-PCR primer sequences

Gene (human)	Primers (5'-3')	Product size (bps)
HAVCR2	F: CTGCTGCTACTACTTACAAGGTC<br>R: GCAGGGCAGATAGGCATTCT	75
GAPDH	F: ACAACTTTGGTATCGTGGAAGG<br>R: GCCATCACGCCACAGTTTC	101
CD80	F: AAACTCGCATCTACTGGCAAA<br>R: GGTTCTTGTACTCGGGCCATA	87
CD83	F: AAGGGGCAAAATGGTTCTTTCG<br>R: GCACCTGTATGTCCCCGAG	96
CD86	F: CTGCTCATCTATACACGGTTACC<br>R: GGAAACGTCGTACAGTTCTGTG	133
HLA DRA	F: AGTCCCTGTGCTAGGATTTTTCA<br>R: ACATAAACTCGCCTGATTGGTC	131
B2M	F: GAGGCTATCCAGCGTACTCCA<br>R: CGGCAGGCATACTCATCTTTT	248
CCR7	F: ATTTGTTTCGTGGGCCTACTG<br>R: TCATGGTCTTGAGCCTCTTGA	76
CD14	F: ACGCCAGAACCTTGTGAGC<br>R: GCATGGATCTCCACCTCTACTG	122
CCL17	F: TTCTCTGCAGCACATCCACGCA<br>R: CTGGAGCAGTCCTCAGATGTCT	129
CCL18	F: GTTGACTATTCTGAAACCAGCCC<br>R: GTCGCTGATGTATTTCTGGACCC	120
CD11c	F: AGAGCTGTGATAAGCCAGTTCC<br>R: AATTCCTCGAAAGTGAAGTGTGT	95
CD74	F: GATGACCAGCGCGACCTTATC<br>R: GTGACTGTCAGTTTGTCCAGC	200
HLA-DQA1	F: TCGCTCTGACCACCGTGAT<br>R: AGGGACCGTAAAACTGGTACAA	99
HLA-DQA2	F: TGCCTCCTATGGTGTGAACTT<br>R: AGACAGTCTCTTTCGTCTCCAG	113
HLA-DQB1	F: ACCTTCGGGTAGCAACTGTC<br>R: AAATCCTCGGGAGAGTCTCTG	82

HLA-DPB1	F: CAGCACCACAACCTGCTTG<br>R: CCATTCAGGAACCATCGGACT	77
HLA-DPA1	F: ATGCGCCCTGAAGACAGAATG<br>R: ACACATGGTCCGCCTTGATG	112
HLA-DRB9	F: TGGAGCAGATTAAACACGAGTG<br>R: CCGCCCGGAACTTTCTGAC	125
